# Hemodynamic Responses to Low-Load Blood Flow Restriction and Unrestricted High-Load Resistance Exercise in Older Women

**DOI:** 10.3389/fphys.2018.01324

**Published:** 2018-10-01

**Authors:** Brendan R. Scott, Jeremiah J. Peiffer, Hannah J. Thomas, Kieran J. Marston, Keith D. Hill

**Affiliations:** ^1^School of Psychology and Exercise Science, Murdoch University, Perth, WA, Australia; ^2^School of Human Sciences, University of Western Australia, Perth, WA, Australia; ^3^School of Physiotherapy and Exercise Science, Curtin University, Perth, WA, Australia

**Keywords:** strength training, skeletal muscle, blood pressure, cardiovascular, rating of perceived exertion

## Abstract

**Introduction:** Blood flow restriction (BFR) during low-load resistance exercise increases muscle size similarly to high-load training, and may be an alternative to lifting heavy weights for older people at risk of sarcopenia. However, few studies have addressed the safety of such exercise in older people, or whether this is impacted by the actual exercises performed during training. This study aimed to compare the acute hemodynamic and perceptual responses during low-load BFR exercise to unrestricted low-load and high-load exercise in older women, and to determine whether these responses depend on the type of exercise performed.

**Methods:** Fifteen older women (63–75 year) were assessed for maximal strength (1RM) in the leg press and leg extension. Participants then completed three protocols using these exercises in a randomized order: (1) low-load exercise (LL); (2) low-load exercise with BFR (LL_BFR_), and; (3) high-load exercise (HL). Blood pressure was assessed at baseline and after each set, and impedance cardiography measured cardiovascular function during trials. Rating of perceived exertion (RPE) and muscle soreness scores were obtained throughout trials.

**Results:** Baseline hemodynamic values were consistent between trials. Systolic, diastolic, and mean arterial pressures were higher in LL_BFR_ compared with HL and LL (*p* ≤ 0.021). The LL condition resulted in lower heart rate (*p* ≤ 0.002) and rate-pressure product (*p* ≤ 0.011) responses compared with LL_BFR_ and HL. The leg press generally conferred greater hemodynamic and perceptual demands than the leg extension for all conditions (*p* ≤ 0.002). RPE was lower during LL compared with LL_BFR_ and HL (*p* ≤ 0.008), and there were no between-condition differences in perceived muscle soreness.

**Conclusion:** The blood pressure data indicate that LL_BFR_ causes greater stress on the vasculature than LL and HL exercise, and that the leg press was generally more demanding than the leg extension. While additional cardiovascular measures were similar between LL_BFR_ and HL conditions, caution should be advised when prescribing BFR exercise for individuals with compromised cardiac or vascular function. Nevertheless, LL_BFR_ and HL exercise were perceived similarly, indicating that BFR training may be viable for healthy older people.

## Introduction

Sarcopenia is associated with increased risk of osteoporosis, cardiovascular complications, and decreases in functional independence and impaired performance in tasks of daily living (Marcell, 2003). These factors can result in a sedentary lifestyle, further exacerbating functional declines and increasing the likelihood of falls (Benichou and Lord, 2016). As such, techniques focused on delaying, stopping or reversing the age-related loss of muscle mass should be emphasized for clinicians working with these populations. Guidelines for older adults state that increasing muscle size and strength requires training with weights of ≥60% 1-repetition maximum (1RM), with many individuals requiring a gradual progressive overload to reach these weights (Ratamess et al., 2009). However, such progressions could take several months, increasing the likelihood of program attrition. Furthermore, musculoskeletal conditions that are common in an aging population (e.g., osteoarthritis, gout, back pain) can compromise strength and/or joint stability, resulting in an inability to safely perform exercise with heavy weights (Cook et al., 2017). Low-load resistance exercise with blood flow restriction (BFR), which utilizes inflatable cuffs around the top of the exercising limbs to occlude venous return but maintain arterial inflow (Scott et al., 2015a), represents an alternative training modality for an aging population.

Resistance training with BFR has been shown to enhance muscular size and strength for older adults in several studies (Karabulut et al., 2010; Vechin et al., 2015; Yasuda et al., 2016; Cook et al., 2017). These adaptations are observed when using BFR while lifting light weights (20–40% 1RM) that impose less mechanical stress than traditional training, and would otherwise not normally cause muscular development (Karabulut et al., 2010). Such findings have led researchers to advocate for BFR training to attenuate age-related declines in muscle mass and strength (Scott et al., 2015a). Irrespective of the potentially beneficial functional outcomes (Cook et al., 2017), the use of this technique by clinicians is still somewhat limited due to safety concerns (Spranger et al., 2015). Adding BFR during low-load resistance exercise in older adults can cause acute increases in systolic (SBP) and diastolic (DBP) blood pressure (Vieira et al., 2013; Staunton et al., 2015; Pinto and Polito, 2016), heart rate (HR) (Vieira et al., 2013; Staunton et al., 2015; Pinto and Polito, 2016), systemic vascular resistance (Pinto and Polito, 2016), and rate-pressure product (RPP) (Vieira et al., 2013; Staunton et al., 2015). These responses indicate an additional stress on the vasculature and amplified myocardial workload, prompting the need for caution when prescribing BFR training for older adults with hypertension, vascular dysfunction or heart disease.

The findings of possibly increased stress to the vasculature and myocardium during BFR are important; however, the current data may not accurately reflect normal resistance training in an aged population. No research has described the hemodynamic responses to different types of resistance exercise as they would be typically prescribed in a training session for older adults. The 45° leg press exercise (where the legs are raised above the level of the heart) is a common inclusion in training programs for older adults; yet, the incline position may alter the hemodynamics of this exercise when compared with more upright exercise such as the seated leg extension (where the legs are positioned below the level of the heart), despite both exercises primarily targeting the quadriceps femoris. Indeed, Staunton et al. (2015) have proposed that elevation of the legs during the 45° leg press may assist venous return, which could potentially mitigate increases in systemic vascular resistance in comparison with other exercises. The leg press also involves contribution from the hip extensor muscles, whereas the leg extension does not, which would likely increase the hemodynamic demands of exercise. In addition, few studies have examined the hemodynamic responses to BFR resistance exercise in older women. To our knowledge, the only published research in this population has recruited hypertensive patients (Pinto and Polito, 2016; Pinto et al., 2018), who may be contraindicated for BFR exercise (Kacin et al., 2015) and would therefore be unlikely to engage in this training stimulus. Finally, it is also important to consider how demanding participants perceive this training to be in comparison with more traditional heavy weights training. Adherence to exercise regimes has been identified as a major barrier for those prescribing training to older people (Room et al., 2017), and compliance is likely to decline if exercise is perceived as too difficult.

If BFR training is to become a more widely utilized training modality for older adults, it is imperative to understand the physiological impacts and potential for adverse events which may result from this exercise, as well as how this exercise is perceived by participants. Furthermore, it is also imperative to determine how low-load BFR exercise compares with more traditional heavy training. Should low-load BFR training become a viable alternative to lifting heavy weights for older people, comparisons between these two training structures are important to produce ecologically valid conclusions that can inform practice. The aims of this study were to determine the impacts of BFR on hemodynamic and perceptual responses during two different low-load resistance exercises within a training session, and compare these responses to traditional higher-load exercise.

## Materials and Methods

### Participants

Fifteen older women (aged 63–75 years; **Table 1**) volunteered to participate in this study. Prior to commencement of the study, all participants were provided with information detailing the purpose and requirements of the research, and were screened for medical contraindications using a modified questionnaire designed specifically for BFR exercise (Kacin et al., 2015). All participants were non-smokers, had not undertaken structured resistance training within the previous 6 months, were not taking hormone replacement therapy, and did not present with any musculoskeletal, neurological, or vascular disease/injury. Participants were excluded if they presented with diabetes mellitus, hypertension, a history of blood clotting, or lymphedema. The study and its methods were approved by the Murdoch University Human Ethics Committee, and conducted in accordance with the Declaration of Helsinki. All participants gave written informed consent prior to commencing the study.

**Table 1:** Characteristics of participants at baseline.

Variable	Mean ± SD	Range
Age (year)	66.8 @ 3.8	63–75
Body mass (kg)	65.8 @ 14.6	52.6–106.6
Height (m)	1.64 @ 0.06	1.53–1.75
BMI (kg m^2^)	24.2 @ 4.4	18.8–34.8
SBP (mmHg)	120.2 @ 13.5	99–145
DBP (mmHg)	69.3 @ 7.4	57–89
MAP (mmHg)	86.3 @ 8.8	73.3–108.0
PP (mmHg)	51.0 @ 9.2	35–64
Leg Press 1RM (kg)	85.5 @ 26.8	52.5–150.0
Leg Extension 1RM (kg)	29.8 @ 5.8	21.3–38.8

*BMI, body mass index; SBP, systolic blood pressure; DBP, diastolic blood pressure; MAP, mean arterial pressure; PP, pulse pressure; 1RM, 1-repetition maximum. Values are presented as mean ± SD*.

### Experimental Design

Participants reported to the laboratory at the same time of day on four occasions, each separated by 4–10 days. During their first visit, participants were familiarized with the rating of perceived exertion (RPE) and visual analog scales used in the research. They were then instructed on appropriate technique for the 45° leg press and the leg extension exercises, before performing 1RM testing following protocols described previously (Scott et al., 2015b). Briefly, participants 1RM were defined as their heaviest completed repetition, and was determined within 3–6 attempts. Using a crossover design, participants then visited the laboratory on three additional occasions to complete exercise trials in a randomized order: (1) low-load resistance exercise (LL), (2) low-load resistance exercise with BFR (LL_BFR_), and (3) high-load resistance exercise (HL). During exercise trials, participants were monitored for hemodynamic function via blood pressure and automated impedance cardiography assessments. Participants also provided perceptual responses during and at 24 h following trials, to indicate perceived exertion and muscle soreness.

### Exercise Trials

Upon arriving at the laboratory for exercise trials, participants rested quietly in a recumbent position for 15 min. Participants then warmed-up with 5 min of cycling at 60 RPM at a self-selected resistance, before commencing their assigned exercise protocol. During low-load trials, participants performed three sets of leg press and leg extension exercises with 20% 1RM, including 1 set of 20 repetitions followed by 2 sets of 15 repetitions, with 30 s recovery between sets and 8 min between exercises. During the high-load trial, participants performed 3 sets of 10 repetitions with 70% 1RM for each exercise, resting for 60 s between sets and 8 min between exercises. The low-load and high-load protocols were deliberately not matched for volume load (sets × repetitions × weight), in order to provide ecologically valid comparisons between how these strategies would actually be implemented in a training program (Bird et al., 2005). Similarly, the order of exercises was not randomized to reflect how these two exercises would be prescribed within a single training session; multi-joint exercises recruiting more muscle mass are recommended to be performed before single-joint exercises which utilize less muscle mass (Bird et al., 2005).

### Determination and Implementation of BFR Pressure

Prior to LL_BFR_ trials, arterial occlusion pressure was measured to the nearest 10 mmHg with participants lying in a recumbent position using a handheld bi-directional ultrasound Doppler probe placed on the posterior tibial artery (MD6 Doppler, Hokanson, Bellevue, WA, United States) in accordance with established methods in BFR research (Loenneke et al., 2012). Restriction was applied to the proximal portion of the right thigh using a pressurized cuff (10 cm wide) connected to an E20 rapid cuff inflator and AG101 air source (Hokanson, Bellevue, WA, United States). During LL_BFR_ exercise, restrictive pressure was set to 50% of each participant’s individualized arterial occlusion pressure. The cuffs were inflated for the duration of each exercise (including during inter-set rest periods), but were deflated between exercises.

### Hemodynamic Responses

Measurements of HR, cardiac output (CO), and stroke volume (SV) were obtained during exercise at a beat-by-beat frequency and reported as 10 s mean values using automated impedance cardiography (Q-Link PhysioFlow PF-07, Manatec Biomedical, France). This non-invasive technology involves the application of electrodes to the neck and thorax (as per the manufacturer’s instructions), from which the *trans*-thoracic bio impedance across the cardiac cycle and the electrical activity of the heart (i.e., electrocardiography) can be measured, to quantify hemodynamic variables. This method has been shown to be valid and reliable at rest and during submaximal exercise in patients with normal cardiorespiratory function (Charloux et al., 2000). Manual recordings of SBP and DBP were taken using a standard upper arm cuff and stethoscope (ALP K2, Tanaka Sangyo Co., Ltd., Japan; 12 cm wide internal bladder), at 1 min prior to commencing leg press and leg extension protocols, as well as immediately following the conclusion of each set of exercise. For all three conditions, these blood pressure measurements were obtained from the right arm, using the same equipment, and with the participant resting on the exercise equipment (for the leg press, participants’ feet were resting on the floor rather than being pressed up against the machine platform). For the LL_BFR_ condition, the BFR cuffs were not inflated during the baseline measurement, but were inflated around participants’ thighs during the post-set assessments. The use of manual recording of SBP and DBP has demonstrated smallest detectable differences of 7.6 and 7.0 mmHg, respectively, during rest conditions (Keavney et al., 2000). These data were used to calculate mean arterial pressure [MAP; calculated as 1/3 (SBP–DBP) + DBP] and pulse pressure (PP; calculated as SBP–DBP). To provide details relevant to the highest potential cardiovascular demands during each set, the peak HR, CO and blood pressure measurements recorded during leg presses and leg extensions were used to calculate the highest potential RPP and total peripheral resistance (TPR) for each experimental session.

### Perceptual Responses

A Category Ratio-10 RPE score was obtained immediately following each set, and a session RPE score was collected at 20 min following each trial to indicate the perceived difficulty of each set and the entire session, respectively. Participants also rated their muscle soreness at 24 h following each trial, by marking a 100 mm visual analog scale at a point between 0 (no soreness) and 100 (maximum soreness) (Kon et al., 2012). To anchor soreness to the lower body muscles, participants were instructed to flex and extend their knees before providing their soreness score.

### Statistical Analyses

All data are represented as mean ± SEM unless indicated otherwise. Prior to analyses, the Shapiro–Wilk test confirmed that data were normally distributed. Following this, dependent variables during exercise (hemodynamic parameters and set RPE values) were compared between experimental trials (LL, LL_BFR_, and HL) and time points (i.e., between sets within an exercise, and between matched sets in the leg press and leg extension) using linear mixed models. Trials and time points were set as fixed factors, and participants were set as random factors. Where a significant main effect or interaction was observed, Fisher’s LSD *post hoc* assessment was used to identify where differences occurred. Session-RPE and muscle soreness scores were also analyzed via linear mixed models, with trials set as fixed factors and participants as random factors, and Fisher’s LSD *post hoc* implemented where a significant main effect was observed. The gradient of the increase in set RPE scores was calculated as ΔY÷ΔX; the change in RPE from set 1 to 3 divided by the change in set number (i.e., 3-1 = 2). These gradient values were calculated for all participants during each trial, and then compared between conditions and exercises via linear mixed models as described above. Effect sizes were calculated to determine the magnitude of differences between all comparisons made for trials and time points as Cohen’s *d_z_* (difference in the mean divided by the standard deviation of the difference; 0.20–0.49 = small effect; 0.50–0.79 = moderate effect; and ≥0.80 = large effect) (Cohen, 1988). Statistical analyses were conducted using SPSS (v24, Chicago, IL, United States), with statistical significance set at *p* ≤ 0.05.

## Results

A high level of adherence was observed in this study with only one participant failing to complete all required sets. In this one individual, only the third set of leg press in the LL_BFR_ condition was not completed. Discussion with this participant revealed that they stopped the set due to a zip on their pants being compressed against their leg by the BFR cuffs, which caused localized pain. The participant was able to re-position the cuffs so that they no longer compressed the zip, and then completed all sets of the leg extension exercise without any further pain.

An interaction was observed for volume load between exercise and protocol (*p* < 0.001), with *post hoc* analyses confirming higher volume load scores for both exercises during the high-load protocol (total volume load = 2421 ± 171 kg) compared with the low-load (total volume load = 1153 ± 82 kg) trials (*p* < 0.001; *d_z_* = 3.19–5.16), as well as higher volume loads for the leg press compared with the leg extension exercise for the high-load (leg press = 1796 ± 146 kg, leg extension = 626 ± 31 kg; *p* < 0.001; *d_z_* = 2.47) and low-load (leg press = 855 ± 69 kg, leg extension = 298 ± 15 kg; *p* < 0.001; *d_z_* = 2.47) protocols.

### Hemodynamic Responses

**FIGURE 1:**
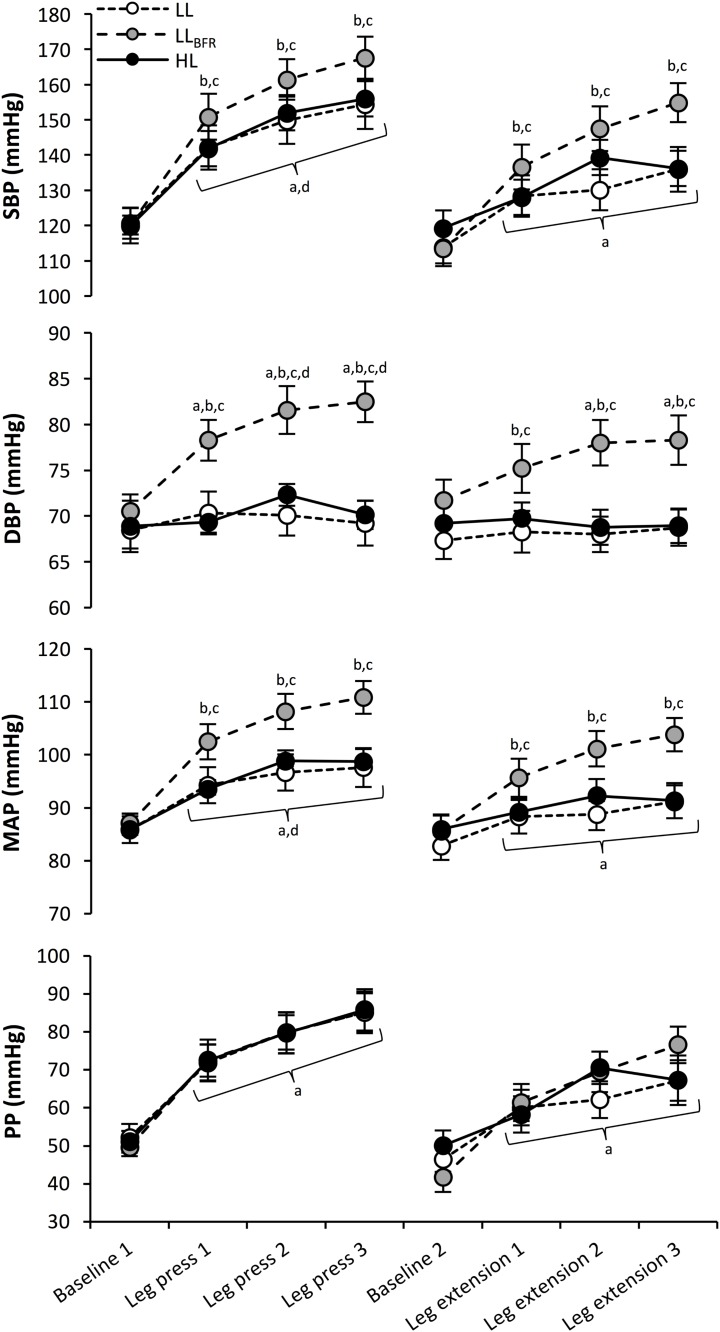
Systolic blood pressure (SBP), diastolic blood pressure (DBP), mean arterial pressure (MAP), and pulse pressure (PP) responses at baseline for each exercise, and immediately following each set (brackets denote differences for all three conditions). ^a^Significantly different to pre-exercise baseline. ^b^Significantly different to HL at the same time point. ^c^Significantly different to LL at the same time point. ^d^Significantly different to leg extension exercise for matched set.

The blood pressure responses for each trial are shown in **Figure 1**. For SBP, DBP, and MAP, significant interactions between time and trial were observed (*p* ≤ 0.02). *Post hoc* analyses determined that SBP was increased from baseline following each set for leg press and leg extension exercise during LL (*p* ≤ 0.002; *d_z_* = 1.54–2.66), LL_BFR_ (*p* < 0.001; *d_z_* = 1.60–3.04), and HL (*p* < 0.001; *d_z_* = 1.52–2.20) trials. SBP was also higher after all sets in the leg press compared to matched sets in the leg extension for all conditions (*p* ≤ 0.001; *d_z_* = 1.10–2.14). Furthermore, LL_BFR_ trials produced higher SBP values after each set compared with both LL (*p* ≤ 0.021; *d_z_* = 1.30–2.15) and HL (*p* ≤ 0.016; *d_z_* = 0.97–1.86) sessions. DBP was increased from baseline only during the LL_BFR_ trial, for each set for leg presses and sets 2–3 for leg extensions (*p* ≤ 0.001; *d_z_* = 1.49–2.77), and was higher after sets 2 and 3 in the leg press compared to matched sets in the leg extension only during LL_BFR_ (*p* ≤ 0.028; *d_z_* = 1.38–1.53). Higher DBP values were also observed during LL_BFR_ compared with LL (*p* ≤ 0.001; *d_z_* = 1.16–2.04) and HL (*p* ≤ 0.004; *d_z_* = 1.21–1.82) for all sets. The MAP responses were similar to SBP, with increases from baseline observed following each set during LL (*p* ≤ 0.030; *d_z_* = 1.35–3.15), LL_BFR_ (*p* < 0.001; *d_z_* = 1.52–3.63), and HL (*p* < 0.037; *d_z_* = 0.96–1.94) trials, and greater values after all sets in the leg press compared to matched sets of the leg extension for all conditions (*p* ≤ 0.031; *d_z_* = 1.24–1.85). Higher values were recorded during LL_BFR_ compared with LL (*p* < 0.001; *d_z_* = 1.17–2.60) and HL (*p* ≤ 0.001; *d_z_* = 1.21–2.07) trials. Furthermore, SBP, DBP, MAP values were not different between HL and LL trials at any point. Regarding PP, a significant main effect was only observed for time (*p* < 0.001), with *post hoc* analyses indicating increases from baseline during exercise were observed for all three trials (*p* < 0.001; *d_z_* = 1.37–2.53), as well as greater values after all sets in the leg press compared to matched sets of the leg extension (*p* ≤ 0.001; *d_z_* = 0.95–1.68).

Additional cardiovascular responses to each exercise trial are presented in **Table 2**. To reflect the periods of greatest cardiovascular demand during exercise, these factors are reported as the peak values calculated during leg press and leg extension exercises. For HR responses, significant main effects were observed for trial and exercise (*p* ≤ 0.001), but not for the interaction between variables. *Post hoc* analyses indicated significantly higher HR during leg press compared with leg extensions across all trials (*p* < 0.001; *d_z_* = 0.83–1.35), and that the LL protocol resulted in significantly lower HR compared with LL_BFR_ (*p* = 0.002; *d_z_* = 1.22–1.51) and HL (*p* = 0.001; *d_z_* = 1.51–1.58) trials. Significant main effects for exercise were observed for CO (*p* < 0.001), with *post hoc* analyses indicating significantly higher CO during leg press compared with leg extensions (*p* < 0.001; *d_z_* = 0.79–1.23). Significant main effects were also observed for exercise in SV (*p* < 0.001). *Post hoc* analyses indicated significantly higher SV during leg press compared with leg extensions (*p* = 0.002; *d_z_* = 0.84–1.21). Regarding RPP calculations, significant main effects were observed for trial and exercise (*p* < 0.001), but not for the interaction between variables. *Post hoc* analyses indicated significantly higher RPP during leg press compared with leg extensions across all trials (*p* < 0.001; *d_z_* = 1.07–1.48). Furthermore, the LL protocol produced significantly lower RPP values compared with LL_BFR_ (*p* ≤ 0.001; *d_z_* = 1.65–2.54) and HL (*p* = 0.011*; d_z_* = 1.16–1.29) trials. For TPR responses, a significant main effect was only noted for exercise (*p* = 0.015), with *post hoc* analyses confirming higher values during the leg extension compared to the leg press (*p* = 0.015; *d_z_* = 1.07–1.36).

**Table 2:** Cardiovascular responses to exercise trials.

	Low-load	Low-load	High-load
		with BFR	
**Leg press**			
*Heart rate (bpm)*	108.9 @ 4.6^a^	115.5 @ 6.0^a,b^	118.7 @ 3.8^a,b^
*Cardiac output (l⋅min^-1^)*	11.7 @ 0.9^a^	13.9 @ 1.9^a^	13.8 @ 1.2^a^
*Stroke volume (ml)*	125.6 @ 8.4^a^	138.4 @ 14.2^a^	135.0 @ 10.4^a^
*Rate-pressure product*	171.2 @ 12.4^a^	192.3 @ 16.9^a,b^	181.7 @ 7.7^a,b^
*Total peripheral resistance*	9.0 @ 0.6^a^	8.9 @ 0.9^a^	7.6 @ 0.6^a^
**Leg extension**			
*Heart rate (bpm)*	101.4 @ 2.3	111.9 @ 4.2^b^	109.1 @ 3.2^b^
*Cardiac output (l⋅min^-1^)*	10.0 @ 0.7	10.4 @ 0.9	10.3 @ 0.5
*Stroke volume (ml)*	106.8 @ 6.6	113.6 @ 15.2	104.6 @ 5.2
*Rate-pressure product*	139.5 @ 7.4	167.2 @ 9.0^b^	156.0 @ 9.3^b^
*Total peripheral resistance*	9.8 @ 0.8	10.6 @ 0.9	9.4 @ 0.4

*^*a*^Significantly different to leg extension within same condition. ^*b*^Significantly different to low-load*.

### Perceptual Responses

**FIGURE 2:**
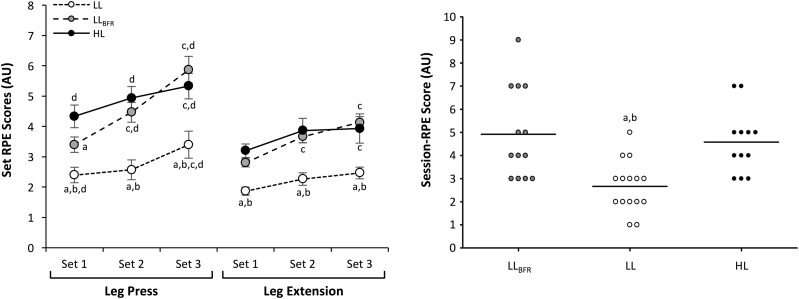
Set and session-rating of perceived exertion (RPE) scores for low-load, low-load with blood flow restriction, and high-load exercise protocols. To provide information regarding individual response to exercise, session-RPE scores have been displayed as individual data points, with mean values indicated by a bold line. ^a^Significantly different to HL. ^b^Significantly different to LL_BFR_. ^c^Significantly different to Set 1 for same exercise. ^d^Significantly different to leg extension exercise for matched set.

The RPE values for each set of exercise and session RPE values for each trial are presented in **Figure 2**. An interaction between trial and time were observed for the RPE of each set during experimental trials (*p* ≤ 0.031). *Post hoc* analyses confirmed that RPE tended to increase across sets for both exercises in each trial (*p* ≤ 0.035; *d_z_* = 0.84–1.68). The set RPE scores were lower for every set during LL compared with LL_BFR_ (*p* ≤ 0.008; *d_z_* = 1.17–1.59) and HL (*p* < 0.001; *d_z_* = 0.97–1.76), while set 1 of the leg press was lower in LL_BFR_ compared with HL (*p* = 0.008; *d_z_* = 0.87). Regarding the gradient of increase in set RPE scores, significant main effects were observed for trial and exercise (*p* ≤ 0.001), but not for the interaction between variables. *Post hoc* analyses indicated a significantly larger gradient (i.e., rate of increase in RPE) during LL_BFR_ compared to LL (*p* < 0.001; *d_z_* = 1.04–1.06) and HL (*p* < 0.001; *d_z_* = 1.08–1.17). Furthermore, RPE increased faster for the leg press compared with the leg extension (*p* < 0.001; *d_z_* = 1.01–2.84).

For session RPE, a main effect was observed for condition (*p* < 0.001), with LL resulting in lower scores compared with LL_BFR_ (*p* < 0.001; *d_z_* = 1.68) and HL (*p* < 0.001; *d_z_* = 0.93). There were no differences in session RPE between LL_BFR_ and HL. Perceived muscle soreness at 24 h post exercise was low for all conditions (HL = 10.8 ± 7.4 mm, LL_BFR_ = 9.4 ± 4.1 mm, and LL = 1.4 ± 0.7 mm), with no main effect for condition observed. Unfortunately, not all participants provided a session-RPE score following the LL_BFR_ (13 responses) or the HL (12 responses) trials, and similar problems were encountered in obtaining muscle soreness values at 24 h following the LL (14 responses), LL_BFR_ (14 responses), and HL (12 responses) trials.

## Discussion

The main findings of this study indicate that, (1) adding BFR to light-weight resistance exercise increases SBP, DBP, and MAP to values which exceed those observed during traditional higher-load exercise, (2) LL_BFR_ and HL exercise resulted in similar HR responses and myocardial workload, which were greater than during LL exercise, (3) LL_BFR_ and HL were perceived to be of similar difficulty, while LL was reported as less strenuous, and (4) the leg press was more demanding than the leg extension, and these effects may be exaggerated by BFR. These findings provide new insights regarding the safety of LL_BFR_ training for older women; while this population has been proposed to benefit from a BFR training approach (Cook et al., 2017), very few studies have examined cardiovascular responses in these individuals or the differences between different types resistance exercises when combined with BFR.

While it may be expected that higher-load exercise could exacerbate blood pressure responses (Williams et al., 2007), in the current study we observed similar SBP, DBP, and MAP responses in the LL and HL protocols. These findings are likely due to the different number of repetitions performed between light and heavy training sessions. For example, Polito et al. (2007) demonstrated similar blood pressure responses between heavy weights training with few repetitions compared with lighter weights training for more repetitions. Importantly, LL_BFR_ resulted in elevated blood pressures when compared with LL and HL, findings similar to those previously reported for older hypertensive (Pinto and Polito, 2016) and normotensive (Vieira et al., 2013; Sardeli et al., 2017) individuals, as well as for healthy young men (May et al., 2017). These findings have practical relevance for practitioners, especially if they are considering the implementation of BFR training in hypertensive patients. Nevertheless, the observed increases in blood pressures were well below those previously associated with hemorrhagic events (Haykowsky et al., 1996), and consistent with commonly observed changes in blood pressure during graded exercise tests (Olson et al., 2012).

The HR responses in this study indicate that the addition of BFR to the LL exercise confers greater cardiovascular work for the same mechanical stimulus. These findings are consistent with previous reports investigating BFR exercise in older people (Vieira et al., 2013; Staunton et al., 2015; Pinto and Polito, 2016), and are likely the result of an elevated chemoreflex (Victor and Seals, 1989) in response to the well-documented increases in metabolic stress during BFR exercise (Suga et al., 2012; Vieira et al., 2013; Spranger et al., 2015). Interestingly, the elevated HR during LL_BFR_ was comparable to that observed during HL. Similar results have been observed by Pinto et al. (2018), who reported comparable responses to resistance training with (3 × 10 repetitions with 20% 1RM) and without (3 × 10 repetitions with 65% 1RM) BFR. However, the same research group have also reported that using BFR during 3 × 15 repetitions with 20% 1RM causes elevated HR responses compared with 3 × 8 repetitions with 65% 1RM (Pinto and Polito, 2016). These contrasting findings indicate that HR responses to light and heavy resistance exercise can be influenced by BFR and the repetition volume of each set. As such, it is important to consider that the findings from the current study are relevant to exercise performed for a pre-determined number of repetitions in each set, and not to volitional fatigue. Future research should therefore aim to assess how manipulation of acute exercise variables (e.g., load lifted, number of sets and repetitions, lifting tempo) impacts on the physiological responses to exercise with BFR.

In this study, the heavy and light weight protocols were deliberately not matched for volume load in order to quantify the hemodynamic implications when performing these exercises in a typical manner for increasing muscle mass and strength (Scott et al., 2015a). Despite the differences in mechanical demands, LL_BFR_ and HL training were perceived to be of similar difficulty, and both were rated as more strenuous than LL. An interesting observation during exercise was that RPE scores for each set increased more quickly across the protocol in LL_BFR_ compared with HL and LL. It is possible that the more rapid increase in perceived intensity during LL_BFR_ is related to the increase in metabolite accumulation, which is known to occur with each set in BFR exercise (Suga et al., 2012). Particularly when BFR is maintained during the inter-set rest periods in the absence of muscular contractions, as was performed in the current study, the venous occlusion caused by cuffs will limit the removal of metabolites from the limb and compound the local metabolite buildup. However, some authors have suggested that perceived exertion is independent of afferent feedback from skeletal muscle during exercise (Marcora, 2009). Similar perceptual responses to those observed in the current study have been previously reported, whereby a progressive increase in RPE was observed during knee extension exercise in unrestricted high-load (4 × 10 with 70% 1RM) and low-load BFR (1 × 30 and 3 × 15 with 20% 1RM, BFR set at 60% arterial occlusion pressure) conditions (Loenneke et al., 2015). Furthermore, a trend for more rapid increases in RPE during low-load BFR exercise (45% increase from set 1 to 4), compared to during high-load exercise (25% increase from set 1 to 4) was observed (Loenneke et al., 2015). Collectively, these findings indicate that low-load BFR exercise is perceived as similar in difficulty to high-load exercise if ≤4 sets are performed, but could potentially be perceived as more difficult if a substantially greater number of sets are undertaken.

Similar to set RPE scores, session RPE was lowest following the LL condition; yet, comparable between LL_BFR_ and HL. Furthermore, very low levels of muscle soreness with no differences between protocols were observed at 24 h after exercise for all three exercise methods. These data indicate that older women tolerate LL_BFR_ training similarly to more traditionally prescribed higher-load exercise. Considering that perceived difficulty is often cited as a barrier to undertaking resistance training for older adults (Fisher et al., 2017), it appears that adherence to a LL_BFR_ training program may be similar to a high-load unrestricted training program. It must be acknowledged, however, that there are currently no long-term studies which have examined the adherence rates of LL_BFR_ training programs compared with more traditional exercise prescription. If BFR training is to become a viable training strategy for attenuating sarcopenia in older adults, this gap in scientific understanding should be addressed.

Considering the comparison between exercises (i.e., leg extension vs. leg press), higher SBP, MAP, and PP were observed for the leg press compared with matched sets of leg extensions for all conditions. Greater peak HR, CO, SV, and RPP were also observed during the leg press compared with the leg extension. An interesting finding from our study was that DBP was increased in the leg press only during sets 2 and 3 of the LL_BFR_ condition. While this may indicate an exercise-specific response to BFR (i.e., the effects are more pronounced in the leg press than the leg extension), mean values for DBP were <83 mmHg (**Figure 1**), and the magnitude of these changes may not be clinically important. The difference between exercises may be due to the larger muscle mass required to complete the leg press compared with the leg extension, and the position of the participants during the 45° leg press. For instance, during the leg press the active muscle mass is elevated above the heart, necessitating greater force to increase blood flow against the influence of gravity. Furthermore, gravity-enhanced venous return would result in greater central blood volume, and therefore increased SV due to the Frank–Starling mechanism. Importantly, both LL_BFR_ and HL demonstrated greater cardiovascular workload (RPP) compared with LL (main effects). Analyses of the rate of change in RPE across sets also indicated that the leg press had a larger gradient of increase in perceived exertion compared with the leg extension (**Figure 2**). This provides evidence that not only was the leg press more physiologically challenging compared to the leg extension, but it was perceived to become more difficult with each set than the leg extension. Taken together, these collective findings indicate that both the addition of BFR and choice of resistance exercise may influence cardiovascular function during resistance training and therefore need to be considered during prescription.

While this study has provided novel insights regarding the hemodynamic responses to different exercises performed with BFR, some limitations with the experiment should be acknowledged. Firstly, while the level of BFR during exercise was individualized to each participant’s resting arterial occlusion pressure, it is possible that the dissimilar postures during the leg press and leg extension caused different changes in blood flow for the same given BFR pressure. An alternative to this would be to measure arterial occlusion pressure in the specific posture required for each individual exercise prior to training. Nevertheless, considering the within-subject design of our experiment, the between condition comparisons reported in this paper would not have been affected by this limitation. Secondly, the manual assessment of blood pressure during this study was limited to the immediately post-set time point and the impedance cardiography data was collected as 10 s mean values. It is possible that the blood pressure responses during each set (i.e., prior to our measurement time point) were different to the responses we measured, or that the 10 s window of each impedance cardiography data point may have blunted the hemodynamic variables assessed. In light of this, we have reported the peak values for several hemodynamic variables in this study to provide an indication of the highest measured physiological demands during each exercise. Nevertheless, future research should aim to investigate hemodynamic responses to BFR exercise in healthy older women further via continuous blood pressure monitoring technologies. Finally, it should be acknowledged that not all participants provided perceptual responses to the research team after the conclusion of their trials. This was taken into account with the analyses conducted, as linear mixed models are better able to accommodate missing data points than common analysis of variance methods.

## Conclusion

The findings from this study indicate that LL_BFR_ caused greater blood pressures than more traditional heavy training, despite much lower mechanical demands and other cardiovascular and perceptual responses being similar between LL_BFR_ and HL conditions. Interestingly, the leg press exercise generally conferred greater cardiovascular and perceptual responses than the leg extension, which may even be exaggerated by the application of BFR (particularly for DBP). While BFR training with light weights could be beneficial for older people to attenuate sarcopenia without heavy resistance training, our results suggest that caution should be taken if implementing BFR for individuals with hypertension or reduced myocardial ischemic thresholds. For these individuals, smaller muscle mass exercises such as leg extensions may not increase hemodynamic stress as much as a leg press, though the practitioner should also be aware that a single-joint exercise may have more limited transfer to multi-joint tasks of daily living.

## Author Contributions

BS, JP, and KH developed the concept for this research project. HT performed all recruitment and screening. HT and KM performed all data collection. BS and KM performed data and statistical analyses. BS drafted the original manuscript. All authors made significant contributions to editing and finalizing the manuscript and also approved the final version of the manuscript.

## Conflict of Interest Statement

The authors declare that the research was conducted in the absence of any commercial or financial relationships that could be construed as a potential conflict of interest.
